# Generation of Leukaemia-Derived Dendritic Cells (DC_leu_) to Improve Anti-Leukaemic Activity in AML: Selection of the Most Efficient Response Modifier Combinations

**DOI:** 10.3390/ijms23158333

**Published:** 2022-07-28

**Authors:** Christoph Schwepcke, Lara Kristina Klauer, Diana Deen, Daniel Christoph Amberger, Zuzana Fischer, Fatemeh Doraneh-Gard, Carina Gunsilius, Annika Hirn-Lopez, Tanja Kroell, Johanna Tischer, Melanie Weinmann, Jan-Ole Werner, Andreas Rank, Christoph Schmid, Helga Maria Schmetzer

**Affiliations:** 1 Department of Medicine III, University Hospital of Munich, 81377 Munich, Germany; christoph.kugler@med.uni-muenchen.de (C.S.); lara.klauer@med.uni-muenchen.de (L.K.K.); diana.deen@med.uni-muenchen.de (D.D.); daniel.amberger@med.uni-muenchen.de (D.C.A.); zuzana.fischer@med.uni-muenchen.de (Z.F.); fatemeh.doranehgard@med.uni-muenchen.de (F.D.-G.); carina.gunsilius@med.uni-muenchen.de (C.G.); annika.hirn@med.uni-muenchen.de (A.H.-L.); tanja.kroell@med.uni-muenchen.de (T.K.); johanna.tischer@med.uni-muenchen.de (J.T.); melanie.weinmann@med.uni-muenchen.de (M.W.); 2 Department of Haematology and Oncology, University Hospital of Tuebingen, 72076 Tuebingen, Germany; jan-ole.werner@klinikum-nuernberg.de; 3 Department of Haematology and Oncology, University Hospital of Augsburg, 86156 Augsburg, Germany; andreas.rank@uk-augsburg.de (A.R.); christoph.schmid@uk-augsburg.de (C.S.)

**Keywords:** leukaemia derived DC, acute myeloid leukaemia, anti-leukaemic functionality

## Abstract

Dendritic cells (DC) and leukaemia derived DC (DC_leu_) are potent stimulators of anti-leukaemic activity in acute myeloid leukaemia (AML) and can be generated from mononuclear cells in vitro following standard DC/DC_leu_-generating protocols. With respect to future clinical applications though, DC/DC_leu_-generating protocols specifically designed for application in a whole-blood-(WB)-environment must be established. Therefore, we developed ten new DC/DC_leu_-generating protocols (kits; Kit-A/-C/-D/-E/-F/-G/-H/-I/-K/-M) for the generation of DC/DC_leu_ from leukaemic WB, containing calcium-ionophore, granulocyte-macrophage-colony-stimulating-factor (GM-CSF), tumour-necrosis-factor-alpha, prostaglandin-E_1_ (PGE_1_), prostaglandin-E_2_ (PGE_2_) and/or picibanil (OK-432). All protocols were evaluated regarding their performance in generating DC/DC_leu_ using refined classification and/or ranking systems; DC/DC_leu_ were evaluated regarding their performance in stimulating anti-leukaemic activity using a cytotoxicity fluorolysis assay. Overall, we found the new kits capable to generate (mature) DC/DC_leu_ from leukaemic WB. Through refined classification and ranking systems, we were able to select Kit-I (GM-CSF + OK-432), -K (GM-CSF + PGE_2_) and -M (GM-CSF + PGE_1_) as the most efficient kits in generating (mature) DC/DC_leu_, which are further competent to stimulate immunoreactive cells to show an improved anti-leukaemic cytotoxicity as well. This great performance of Kit-I, -K and -M in mediating DC/DC_leu_-based anti-leukaemic immunity in a WB-environment in vitro constitutes an important and directive step for translating DC/DC_leu_-based immunotherapy of AML into clinical application.

## 1. Introduction

Acute myeloid leukaemia is a malignant disorder of the hematopoietic system, characterised by an uncontrolled proliferation of abnormally differentiated myeloid blasts [1,2]. It is the predominant subtype of leukaemia in adults with a median age of 72 years at diagnosis [3]. Standard treatment options include chemotherapy with or without allogeneic stem cell transplantation (SCT) [4,5], which have an overall 5-year-survival-rate of 29.5% that decreases to only 8.9% for patients over the age of 65 years [6]; the outcome remains unsatisfactory.

Alternative treatment options are needed. Above all, immunomodulatory approaches aiming to (re-)activate the immune system against leukaemia have shown auspicious results [7]. Specifically, dendritic cells (DC) have been put into the limelight of research: DC are one of the most potent mediators of the immune system. As antigen-presenting cells, they have the competence to link the innate and adaptive immune system and mediate a comprehensive immune response to a particular antigen [8,9]. Based on these powerful properties, manipulating DC to present leukaemia-specific antigens proposes to be an auspicious way to induce leukaemia-specific immunity.

There are diverse approaches to manipulating DC: DC can be generated from monocytes in vitro (known as moDC), pulsed with leukaemic peptides [10], apoptotic leukaemic cells or leukaemic cell lysates [11,12], fused with leukaemic blasts [13] or electroporated with messenger ribonucleic acid (mRNA) encoding leukaemia-associated-antigens (LAA) [14] and transferred to the patient as a vaccine. Moreover, DC can be generated from leukaemic blasts in vitro and potentially in vivo (known as DC_leu_), which are characterised by the simultaneous expression of dendritic- and leukaemia-specific antigens [15,16,17,18,19]. The latter not only has the advantage of being unrestricted to a certain LAA, thereby being able to stimulate the immune system to the whole leukaemic antigen repertoire, but also of being less complex as in vivo generation of DC/DC_leu_ circumvents cumbersome manufacturing of LAA-specific moDC under good manufacturing practice (GMP) conditions and administration to the patient.

**Figure 1:**
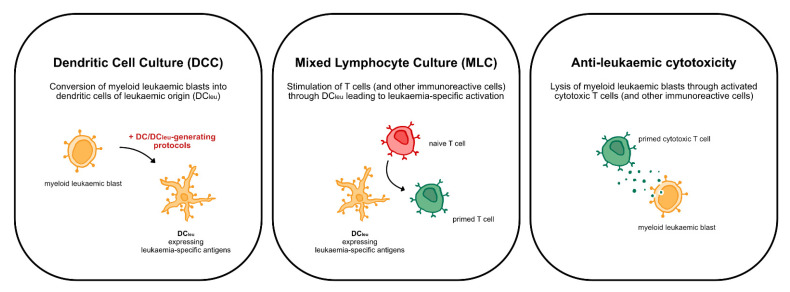
Overview of the concept of DC/DC_leu_-based immunotherapy depicting the steps of generating DC_leu_ through DC/DC_leu_-generating protocols in the dendritic cell culture (DCC) and stimulating T cell enriched immunoreactive cells with generated DC_leu_ in the mixed lymphocyte culture (MLC), overall leading to anti-leukaemic cytotoxicity of T cell enriched immunoreactive cells.

It is well known that myeloid leukaemic blasts can be converted into dendritic cells (DC_leu_) that have the competence to (re-)activate the innate and adaptive immune system against leukaemia [17,18,19,20,21,22,23,24] (Figure 1). This conversion of leukaemic blasts into DC_leu_ can be induced using different sets of response modifiers (DC/DC_leu_-generating protocols) that incorporate the induction of hematopoietic differentiation, dendritic activation and maturation [16]. DC/DC_leu_-generating protocols like Ca, Mcm and Pici have already demonstrated their capacity to generate (mature) DC and DC_leu_ (DC/DC_leu_) from peripheral blood mononuclear cells (MNC) and therewith stimulate an anti-leukaemic immune response in vitro [20,21,25].

With regard to future clinical applications though, more physiological conditions have to be endeavoured, and thus, progression from DC/DC_leu_-generation in an MNC- to a whole-blood-(WB)-environment is inevitable. WB, in contrary to MNC, not only contains the full spectrum of soluble (e.g., cytokines, chemokines) and cellular (e.g., granulocytes, erythrocytes) components that take part in an immune response but also that determine the patient- and tumour-specific environment, consequently influencing the generation of DC/DC_leu_ and the mediation of DC/DC_leu_-based anti-leukaemic immunity.

To ensure efficient DC/DC_leu_-generation in a novel WB-environment, we developed ten new DC/DC_leu_-generating protocols (kits; Kit-A, -C, -D, -E, -F, -G, -H, -I, -K, -M; deduced from Ca, Mcm, Pici), specifically designed for the generation of DC/DC_leu_ from leukaemic WB. Kits were composed of up to three response modifiers, including granulocyte-macrophage-colony-stimulating-factor (GM-CSF), tumour-necrosis-factor-alpha (TNFa), interferon-alpha (IFNa), prostaglandin-E_1_ and -E_2_ (PGE_1_, PGE_2_), picibanil (OK-432) and Calcium-Ionophore A23187 (Ca-Iono). The success of kits in generating DC/DC_leu_ though is strongly dependent on the response modifiers’ (synergistic) potential to induce hematopoietic differentiation, dendritic activation and maturation [16]. Setting up our kits, we thus decided upon GM-CSF, IFNa and Ca-Iono for their potential to induce differentiation of myeloid leukaemic blasts to dendritic cells [26,27,28,29,30], OK-432 for its potential to activate dendritic cells through danger signalling [31,32] and GM-CSF, TNFa, IFNa, OK-432, PGE_1_, PGE_2_ and Ca-Iono for their potential to induce maturation of dendritic cells through CCR7 expression [26,28,33,34,35,36,37]. Ultimately, to determine the best performing kits, in addition to the capacity of kits to generate DC/DC_leu_, the capacity of kit-generated DC/DC_leu_ to stimulate anti-leukaemic immunity must be taken into consideration.

In this study, we developed DC/DC_leu_-generating protocols specifically designed for the generation of DC/DC_leu_ in a WB-environment and evaluated their potential to generate DC/DC_leu_ and mediate DC/DC_leu_-based anti-leukaemic immunity. This constitutes an important and directive step for translating DC/DC_leu_-based immunotherapy into clinical application.

## 2. Results

### 2.1. DC/DC_leu_-Generation Using Standard Protocols Is Comparable from MNC and WB

**Figure 2:**
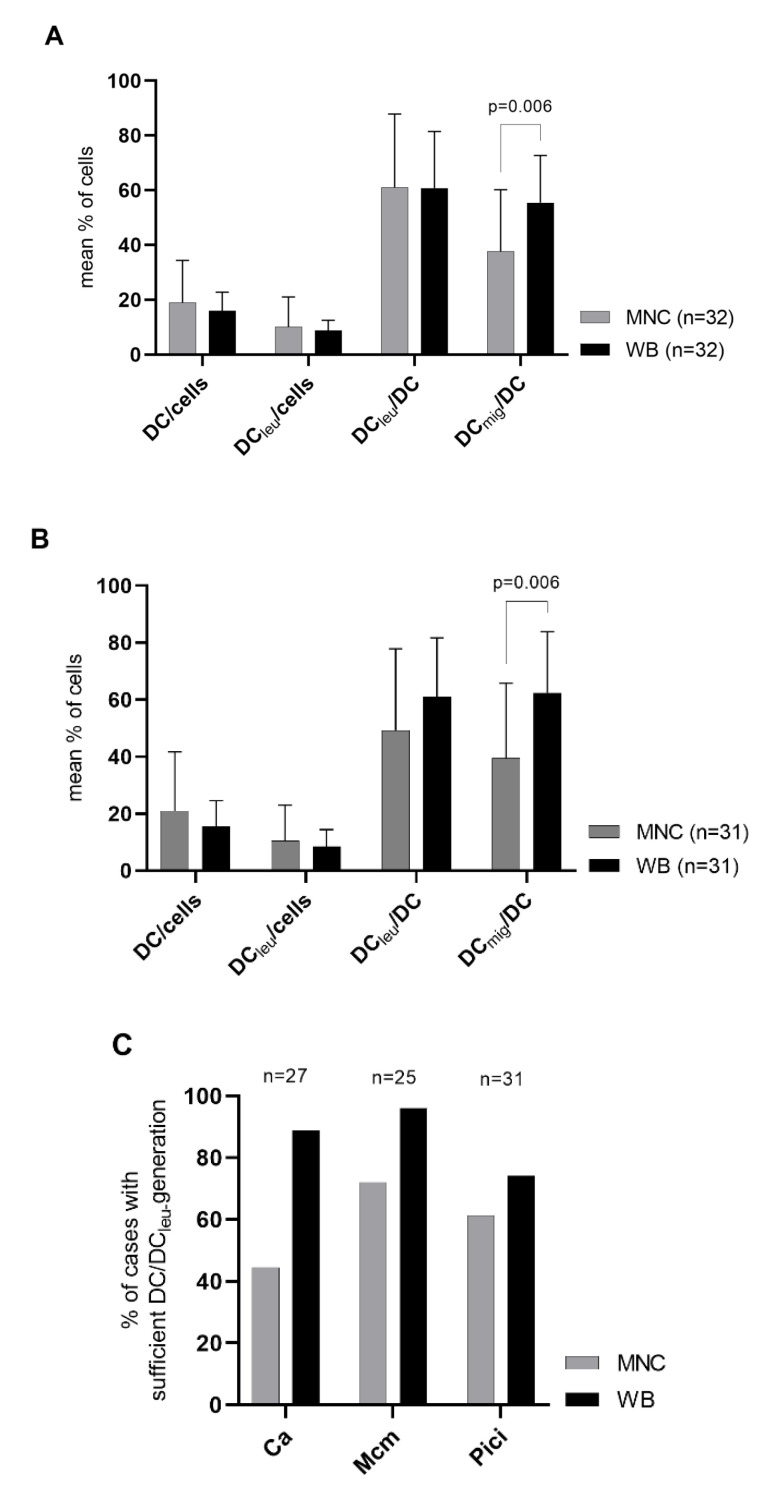
DC/DC_leu_-generation from leukaemic WB and MNC with standard DC/DC_leu_-generating protocols (Ca, Mcm, Pici). Given are the mean ± SD of DC/cells, DC_leu_/cells, DC_leu_/DC and DC_mig_/DC generated with all standard protocols pooled (**A**) and with the standard protocol Pici (**B**) from MNC compared to WB. Further shown are proportions of cases with sufficient DC/DC_leu_-generation following the DC/DC_leu_-classification using standard protocols (Ca, Mcm, Pici) in MNC compared to WB (**C**). Statistically significant differences (*p* values < 0.05) (two-tailed *t*-test) are given.

**Figure A1:**
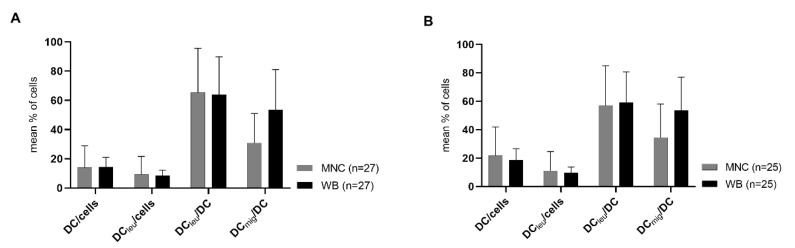
DC/DC_leu_-generation from leukaemic WB and MNC with standard DC/DC_leu_-generating protocols (Ca, Mcm, Pici). Given are the mean ± SD of DC/cells, DC_leu_/cells, DC_leu_/DC and DC_mig_/DC generated with the standard protocol Ca (**A**) and Pici (**B**) from MNC compared to WB. Statistically significant differences (*p* values < 0.05) (two-tailed *t*-test) are given.

We were able to generate significant frequencies of (mature) DC and DC_leu_ from both MNC and WB with standard methods (Ca, Mcm, Pici). Pooling all standard methods, we found no significant differences in frequencies of DC/cells, DC_leu_/cells and DC_leu_/DC comparing MNC- and WB-cultures, but significantly higher frequencies of DC_mig_/DC in WB-cultures compared to MNC-cultures (Figure 2A). Considering all standard methods separately, we found no significant differences in frequencies of DC/cells, DC_leu_/cells and DC_leu_/DC comparing MNC- and WB-cultures but (significantly) higher frequencies of DC_mig_/DC in WB-cultures compared to MNC-cultures (Figure 2B, exemplary shown Pici; Figure A1).

Evaluating proportions of cases with sufficient and insufficient DC/DC_leu_-generation according to the DC/DC_leu_-classification (Table 1), we found higher proportions of cases with sufficient DC/DC_leu_-generation using WB compared to MNC after treatment with all three standard protocols (Figure 2C).

In summary, we found the generation of (mature) DC/DC_leu_ using standard protocols equivalent or even superior in WB compared to MNC. Subsequent experiments were thus performed in a WB-environment simulating more physiological conditions.

### 2.2. DC/DC_leu_-Generation from WB Is Comparable Using New Protocols (Kits) and Standard Protocols

We introduced nine new DC/DC_leu_-generating protocols (kits; Kit-A, -C, -D, -E, -F, -G, -H, -I, -K) and compared their capacity to generate DC and DC_leu_ (subgroups) to the performance of the standard protocols they derived from in a WB-environment. We thus compared results of Kit-A, -C, -E, -G, -K to Mcm (sharing GM-CSF, TNFa and/or PGE_2_), Kit-E, -H to Mcm (all cytokine-based), Kit-D, -G, -I to Pici (sharing GM-CSF and/or OK-432) and Kit-F to Ca (sharing Ca-Iono).

**Figure 3:**
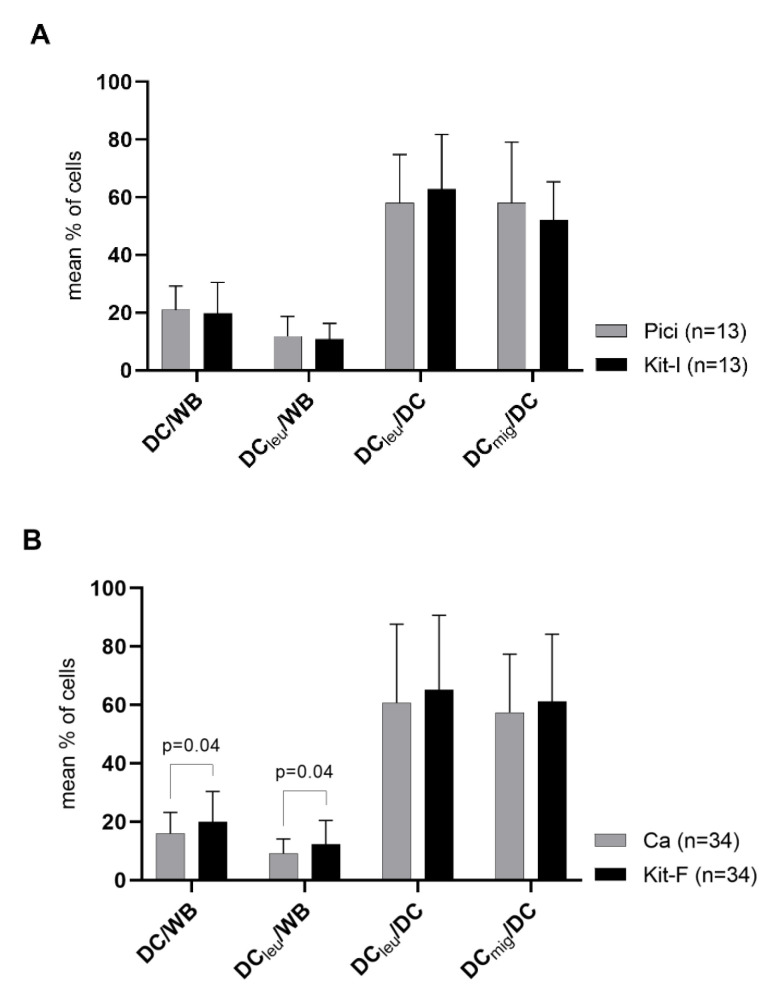

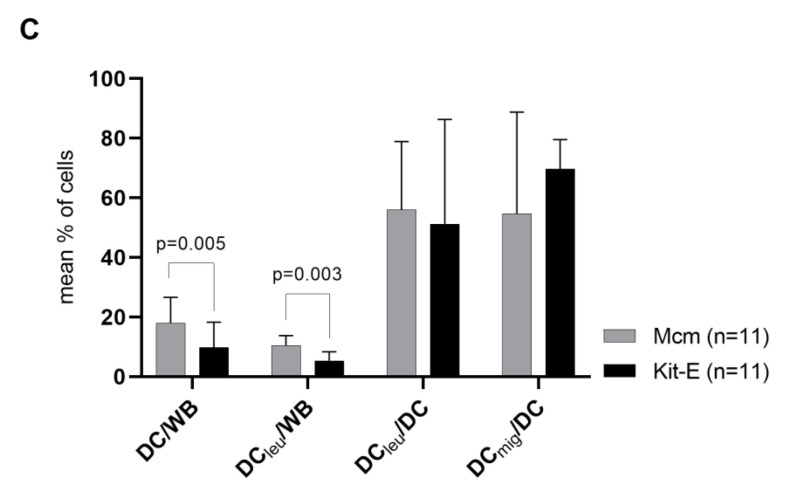
DC/DC_leu_-generation from leukaemic WB with standard (Ca, Mcm, Pici) and new DC/DC_leu_-generating protocols (kits). Given are the mean ± SD of DC/cells, DC_leu_/cells, DC_leu_/DC and DC_mig_/DC generated with Pici compared to Kit-I (**A**), Ca compared to Kit-F (**B**) and Mcm compared to Kit-E (**C**). Statistically significant differences (*p* values < 0.05) (two-tailed *t*-test) are given.

**Figure A2:**
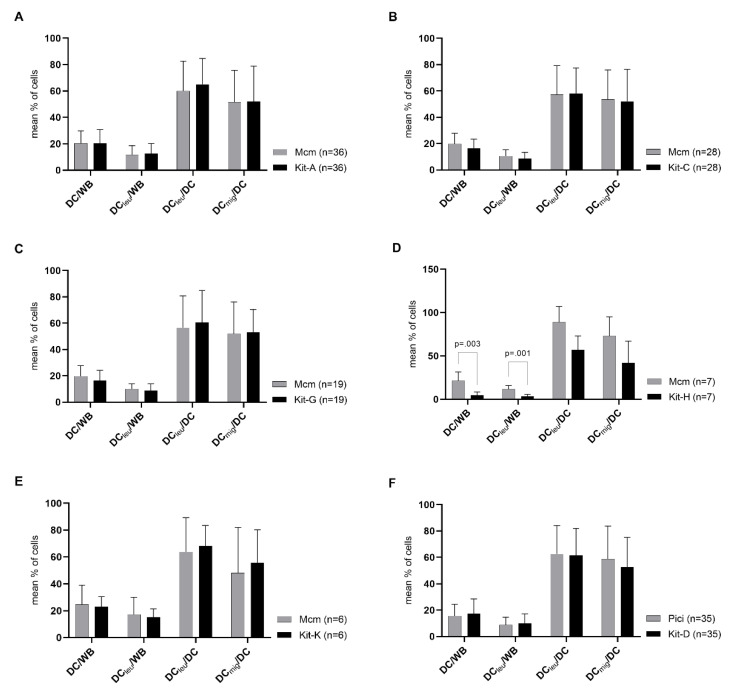

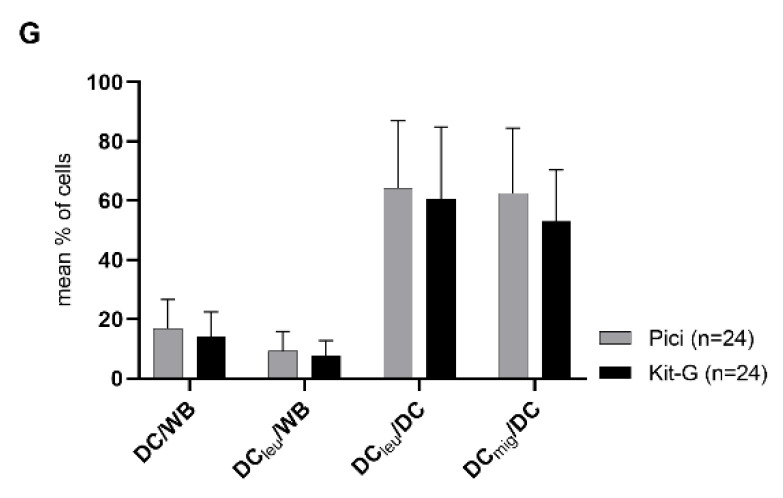
DC/DC_leu_-generation from leukaemic WB with standard (Ca, Mcm, Pici) and new DC/DC_leu_-generating protocols (kits). Given are the mean ± SD of DC/cells, DC_leu_/cells, DC_leu_/DC and DC_mig_/DC generated with Mcm compared to Kit-A (**A**), to Kit-C (**B**), to Kit-G (**C**), to Kit-H (**D**), and to Kit-K (**E**), and Pici compared to Kit-D (**F**) and Kit-G (**G**). Statistically significant differences (*p* values < 0.05) (two-tailed *t*-test) are given.

We found no significant differences in frequencies of DC and DC_leu_ (subtypes) generated with Kit-A, -C, -D, -G, -I, -K compared to standard protocols (Figure 3A, exemplarily shown Kit-I vs. Pici; Figure A2). Kit-F even generated significantly higher frequencies of DC/WB and DC_leu_/WB compared to Ca (Figure 3B). However, Kit-E, -H generated significantly lower frequencies of DC/WB and DC_leu_/WB compared to Mcm (Figure 3C, exemplarily shown Kit-E vs. Mcm; Figure A2). Frequencies of DC_leu_/DC and DC_mig_/DC were comparable between kits and standard protocols.

**Figure 4:**
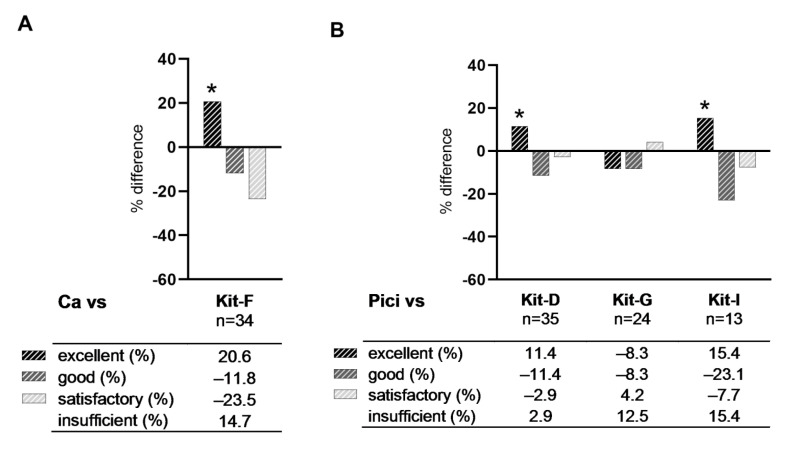

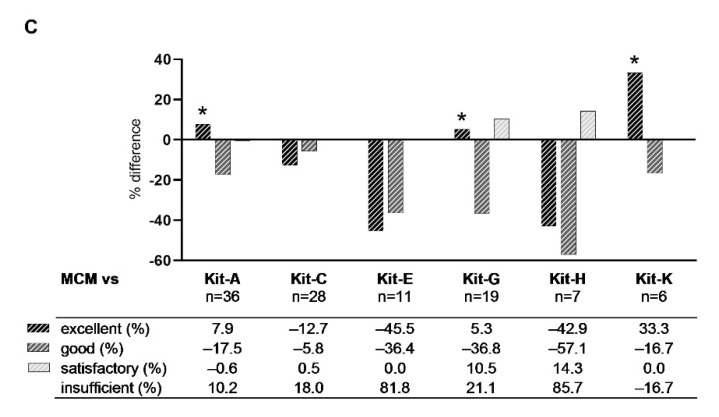
Comparison of DC/DC_leu_-generation from leukaemic WB with standard (Ca, Mcm, Pici) and new DC/DC_leu_-generating protocols (kits). Given are the percentual differences of proportions of cases with sufficient (excellent, good, satisfactory) and insufficient DC/DC_leu_-generation following the DC/DC_leu_-classification when comparing the standard protocol Ca with its derivative protocol Kit-F (**A**), Pici with its derivative protocols Kit-D, -G, -I (**B**), and Mcm with its derivative protocols Kit-A, -C, -E, -G, -H, -K (**C**). An increase of proportions of cases with excellent DC/DC_leu_-generation is marked with *.

Evaluating proportions of cases with sufficient (excellent, good, satisfactory) and insufficient DC/DC_leu_-generation according to the DC/DC_leu_-classification, we found Kit-F (compared to Ca), Kit-D and -I (compared to Pici) and Kit-A, -G and -K (compared to Mcm) generating higher proportions of cases with excellent DC/DC_leu_-generation compared to their respective standard protocol. Proportions of cases with good DC/DC_leu_-generation were lower with all kits, proportions of cases with satisfactory DC/DC_leu_-generation were lower with Kit-A, -D, -F, -I and equal or higher with Kit-C, -E, -G, -H, -K compared to their respective standard protocol (Figure 4).

In summary, most kits were able to generate comparable or, in the case of Kit-F, even higher frequencies of DC/DC_leu_ (subgroups) and higher proportions of cases with excellent DC/DC_leu_-generation compared to their respective standard protocol. Of note, Kit-E and -H were not able to generate comparable frequencies of DC/DC_leu_ (subgroups) but rather generated significantly lower frequencies compared to their standard protocol.

### 2.3. Ranking of Kits

We assessed the potential of kits based on two different ranking methods: (1) the class-ranking based on the proportions of excellent, good, satisfactory and insufficient results of DC/DC_leu_-generation using quantities of generated DC/WB and DC_leu_/WB, and (2) the best-ranking based on the proportions of best and second-best results of DC/DC_leu_-generation using quantities of generated DC/WB.

#### 2.3.1. Class-Ranking of Kits

**Figure 5:**
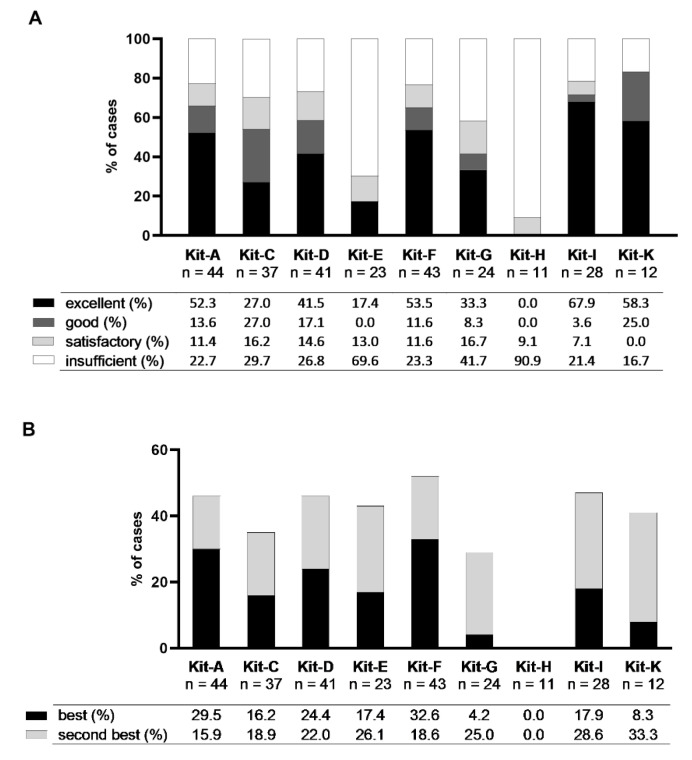
Class- and best-ranking of Kit-A, -C, -D, -E, -F, -G, -H, -I and -K. Given are (**A**) the class-ranking with the proportions of cases with sufficient (excellent, good, satisfactory) and insufficient DC/DC_leu_-generation using kits and (**B**) the best-ranking with proportions of cases with best- and second-best DC/DC_leu_-generation using kits.

We ranked kits based on quantities of generated DC/WB and DC_leu_/WB. We therefore developed three subordinate rankings (ranking 1–3) and one superordinate ranking (ranking 4) (Figure 5A, Table 2). Ranking 1 considers kits based on their proportion of cases with excellent DC/DC_leu_-generation. In doing so, Kit-I and -K performed the best (68% and 58% of cases, respectively), followed by Kit-F, -A, -D, -G, -C, -E and -H. Ranking 2 considers kits based on their proportion of cases with excellent and good DC/DC_leu_-generation. In doing so, Kit-K and -I performed the best (83% and 72% of cases, respectively), followed by Kit-A, -F, -D, -C, -G, -E and -H. Ranking 3 considers kits based on their proportion of cases with sufficient (excellent, good, satisfactory) DC/DC_leu_-generation. In doing so, Kit-K and -I performed the best (83% and 79% of cases, respectively), followed by Kit-A, -F, -D, -C, -G, -E and -H. Ranking 4 considers results of rankings 1–3, creating a superordinate ranking, nominating Kit-K and -I as the best performing kits, followed by Kit-A, -F, -D, -C, -G, -E and -H.

#### 2.3.2. Best-Ranking of Kits

We further ranked kits based on the best and second-best results of quantities of generated DC/WB. We therefore developed two subordinate rankings (ranking 1–2) and one superordinate ranking (ranking 3) (Figure 5B, Table 3). Ranking 1 considers kits based on their proportion of cases with the best DC/DC_leu_-generation. In doing so, Kit-F and -A performed the best (33% and 30% of cases, respectively), followed by Kit-D, -I, -E, -C, -K, -G and -H. Ranking 2 considers kits based on their proportion of cases with the best and second-best DC/DC_leu_-generation. In doing so, Kit-F and -I performed the best (52% and 47% of cases, respectively), followed by Kit-D, -A, -E, -K, -C, -G and -H. Ranking 3 considers results of rankings 1–2, creating a superordinate ranking, nominating Kit-F and -A/-D/-I (the latter equally) as the best performing kits, followed by Kit-E, -K, -C, -G and -H.

In summary, the class-ranking of kits found Kit-K and -I to be the best performing kits, the best-ranking of kits found Kit-F, -A, -D and -I to be the best performing kits. Overall, Kit-I appears to be the most proficient kit in generating DC/DC_leu_.

### 2.4. Selection of Best Kits and Evaluation of Their DC/DC_leu_-Generating and Anti-Leukaemic Performance

#### 2.4.1. Selection of Best Kits

We selected the best performing kits for further evaluation. Ranking of kits found Kit-K, -I, -A, -D and -F to be the most proficient kits. As TNFa and Ca-Iono were no longer approved for human systemic treatment though, we had to exclude Kit-A and -F from further considerations. As Kit-D, composed of three response modifiers (GM-CSF, OK-432, PGE_2_), showed no advantage in generating DC/DC_leu_ compared to Kit-I and -K, composed of two response modifiers (GM-CSF with OK-432 or PGE_2_), we moreover excluded Kit-D from further considerations. Lastly, as parallel studies with PGE_1_ showed PGE_2_ and PGE_1_ to have comparable effects on the generation of DC/DC_leu_, we introduced a new Kit-M, derived from Kit-K, consisting of GM-CSF and PGE_1_, for further evaluation [37].

Overall, we selected Kit-I, -K and -M as the most promising kits and evaluated their capacity to generate (mature) DC/DC_leu_ as well as their potential to initiate anti-leukaemic cytotoxicity.

#### 2.4.2. Kit-I, -K and -M Generate Significantly Higher Frequencies of DC/DC_leu_ Compared to Control

**Figure 6:**
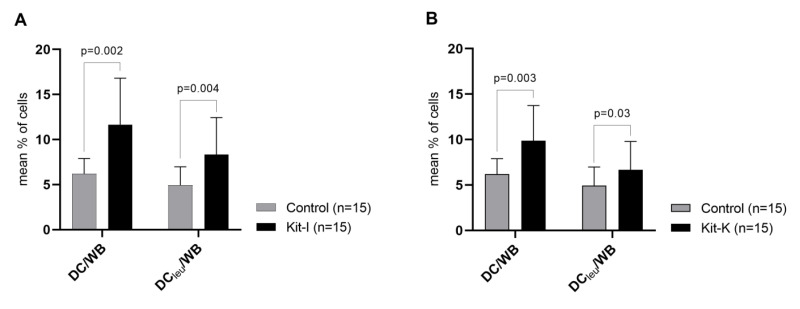

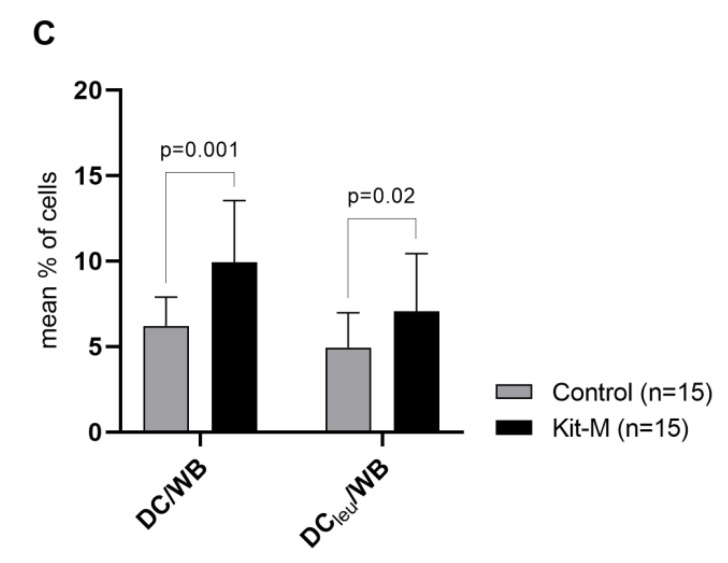
DC/DC_leu_-generation from leukaemic WB with Kit-I, -K and -M. Given are the mean ± SD of DC/cells and DC_leu_/cells generated with Kit-I (**A**), Kit-K (**B**), and Kit-M (**C**) compared to control. Statistically significant differences (*p* values < 0.05) (two-tailed *t*-test) are given.

**Figure 7:**
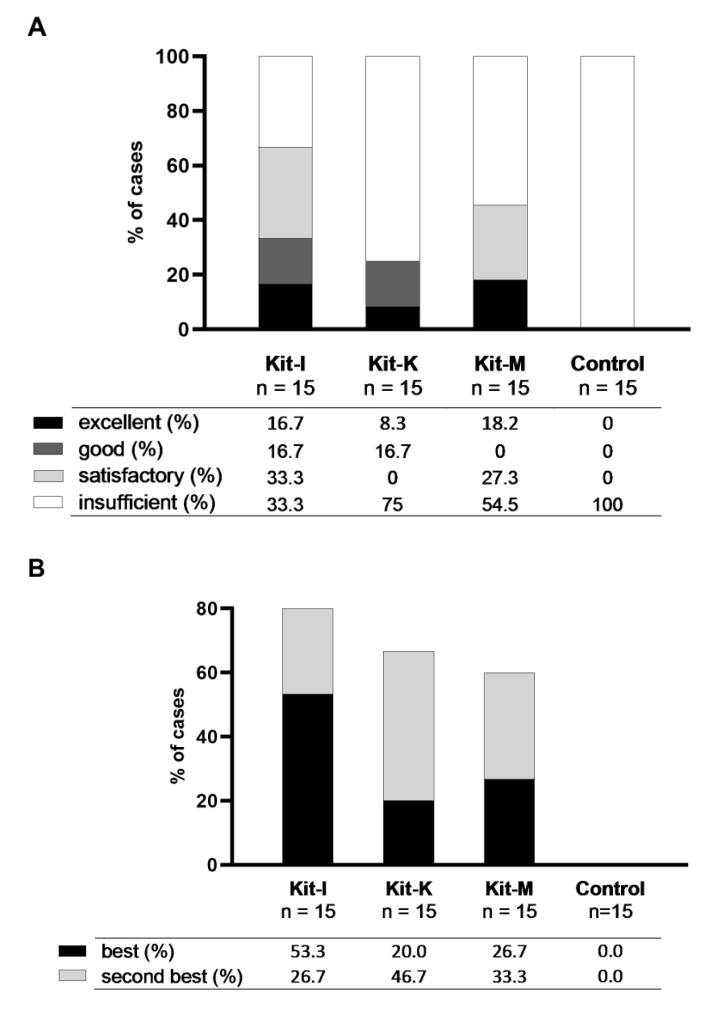
Class- and best-ranking of Kit-I, -K and -M. Given are (**A**) the class-ranking with the proportions of cases with sufficient (excellent, good, satisfactory) and insufficient DC/DC_leu_-generation using kits and (**B**) the best-ranking with proportions of cases with best- and second-best DC/DC_leu_-generation using kits.

All three kits (Kit-I, -K, -M) were able to generate significantly higher frequencies of DC/WB and DC_leu_/WB compared to control (Figure 6). Kit-generated DC/DC_leu_ hereby consisted of significant frequencies of mature DC. Comparing kits among themselves, we found no significant differences in frequencies of generated DC/WB, DC_leu_/WB, DC_leu_/DC and DC_mig_/DC (data not shown). Ranking kits according to the class-ranking and best-ranking, we found Kit-I to be the most efficient kit in generating DC/DC_leu_, followed by Kit-M and -K (Figure 7A,B and Table 4 and Table 5).

#### 2.4.3. DC/DC_leu_ Generated with Kit-I, -K and -M Stimulate Anti-Leukaemic Activity

We analysed the improvement in blast lytic activity of MLC^Kit-I^, MLC^Kit-K^ and MLC^Kit-M^ compared to MLC^Control^ through CTX after 3 h and 24 h of incubation of effector and target cells to assess the anti-leukaemic activity of DC/DC_leu_-stimulated immunoreactive cells.

**Figure 8:**
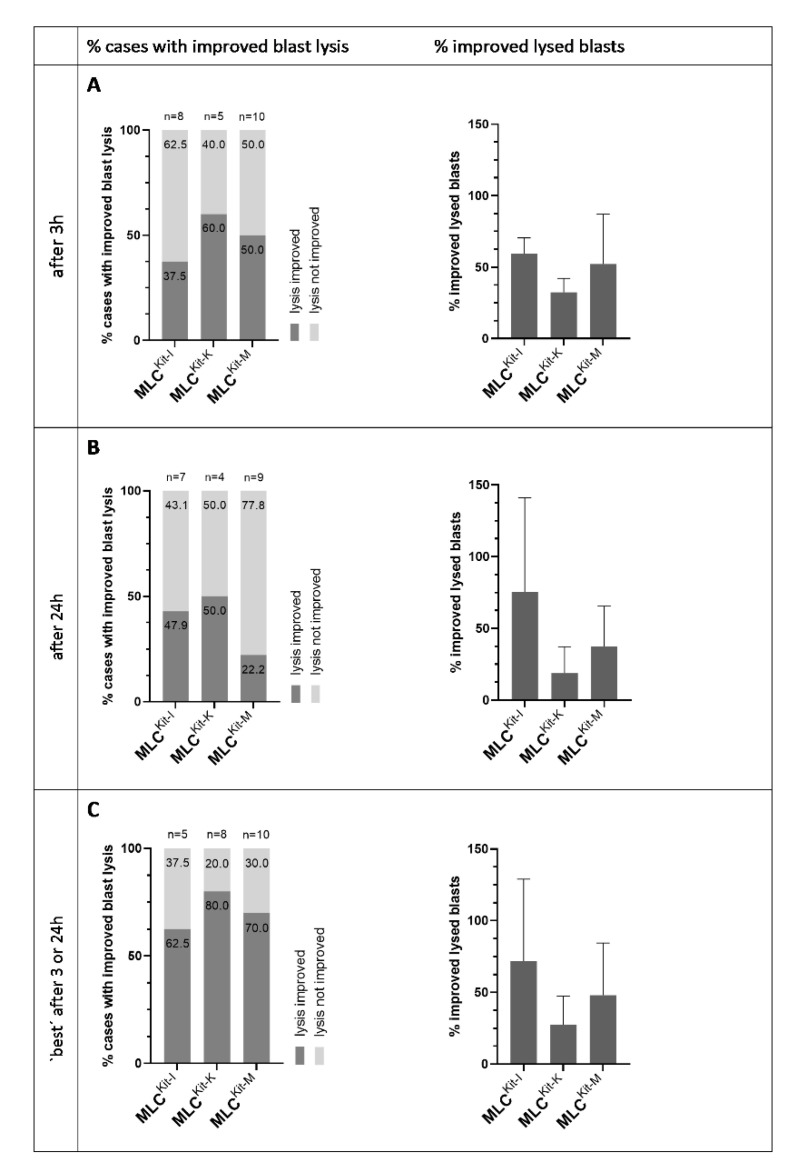
Stimulatory impact of DC/DC_leu_ generated with Kit-I, -K and -M on the anti-leukaemic cytotoxicity of T cell enriched immunoreactive cells. Given are the proportion of cases with an improvement in blast lysis (“% cases with improved blast lysis”) and the mean ± SD of improved lysed blasts (“% improved lysed blasts”) in MLC^Kit-I^, MLC^Kit-K^ and MLC^Kit-M^ in relation to MLC^Control^ after 3 h (**A**) and 24 h (**B**), and the “best” achieved results after 3 h and 24 h (**C**).

After 3 h, we could observe an improvement in blast lysis in about 40% of cases in MLC^Kit-I^, 60% of cases in MLC^Kit-K^ and 50% of cases in MLC^Kit-M^ compared to control. The average improvement in blast lysis was about 60% in MLC^Kit-I^, about 30% in MLC^Kit-K^ and 50% in MLC^Kit-M^ (Figure 8A). After 24 h, we could observe an improvement in blast lysis in about 40% of cases in MLC^Kit-I^, 50% of cases in MLC^Kit-K^ and 20% of cases in MLC^Kit-M^ compared to control. The average improvement in blast lysis was about 75% in MLC^Kit-I^, about 20% in MLC^Kit-K^ and 40% in MLC^Kit-M^ (Figure 8B). Selecting the best-achieved improvement in blast lysis after 3 h or 24 h, we could observe an improvement in blast lysis in about 60% of cases in MLC^Kit-I^, 80% of cases in MLC^Kit-K^ and 70% of cases in MLC^Kit-M^ compared to control. The average improvement in blast lysis was about 70% in MLC^Kit-I^, about 30% in MLC^Kit-K^ and 50% in MLC^Kit-M^ (Figure 8C).

In summary, we found Kit-I, -K and -M gave rise to comparable frequencies of DC/DC_leu_. All three kits were able to improve the anti-leukaemic activity of immunoreactive cells compared to control. Overall, Kit-I, followed by Kit-M and -K, performed the best regarding the class-ranking and best-ranking, with the highest proportions of excellent and best DC/DC_leu_-generation, as well as the best-achieved improvement in blast lysis after 3 h or 24 h. We thus perceive Kit-I and -M to have the highest potential in generating DC/DC_leu_ and stimulating anti-leukaemic activity.

## 3. Discussion

### 3.1. DC_leu_-Based Immunotherapy

DC are one of the most potent mediators of the immune system. Manipulated to express leukaemia-specific antigens, they impose an auspicious way to (re-)activate the immune system to a leukaemia-specific immunity. Particularly, DC generated from myeloid leukaemic blasts (known as DC_leu_), which are characterised by the simultaneous expression of dendritic- and leukaemia-specific antigens, hold the great potential of stimulating the immune system to the whole leukaemic antigen repertoire [15,16,17,18,19]. The process of converting myeloid leukaemic blasts into DC/DC_leu_ in vitro using DC/DC_leu_-generating protocols consisting of various response modifiers has been demonstrated multiple times before [20,21,25]. Most protocols are based on an MNC-setting, though. With respect to future clinical applications, progression to DC/DC_leu_-generation in a WB-setting simulating a more physiological patient- and tumour-specific environment is inevitable. We thus developed new DC/DC_leu_-generating protocols specifically designed for the generation of DC/DC_leu_ from leukaemic WB and evaluated their potential to generate DC/DC_leu_ and mediate DC/DC_leu_-based anti-leukaemic activity.

### 3.2. Standard Protocols—DC/DC_leu_ Generation from Leukaemic MNC and WB

Standard DC/DC_leu_-generating protocols like Ca, Mcm and Pici have been shown to be competent in generating significant frequencies of (mature) DC/DC_leu_ from leukaemic MNC [20,21]. Although their compositions and operations were specifically designed for MNC-application, we nevertheless wanted to examine their performance in a novel WB-environment, simulating more physiological conditions.

We found all standard protocols competent to generate DC/DC_leu_ from MNC and WB to a comparable extent, with higher frequencies of mature DC generated from WB (Figure 2). This is an interesting finding as the stimulation of maturation and CCR7-dependent (lymph node) migration is essential for DC/DC_leu_ to activate T-cells as well as other immunoreactive cells [38,39,40]. DC maturation and migration is a tightly regulated process, controlled by a large variety of chemotactic and non-chemotactic factors like danger-associated-molecular-patterns (DAMPs), acute-phase-proteins (APPs), proinflammatory cytokines or eicosanoids produced by different cell types [41]. A superior maturation of DC/DC_leu_ in a WB- compared to an MNC-environment may be attributed to the fact that WB comprises the full spectrum of soluble (including chemotactic and non-chemotactic) and cellular factors necessary for the homeostasis of the blood and immune system and thus can readily support DC/DC_leu_ maturation and migration. An inferior maturation of DC/DC_leu_ in a (serum-free) MNC-environment in contrary suggests a restriction of this process due to the absence of (readily) available maturation and migration factors. This emphasises the importance of incorporating the full spectrum of soluble and cellular factors present in WB when developing immunomodulatory agents for in vivo use.

Assessing the overall DC/DC_leu_-generating potential of standard protocols using the DC/DC_leu_-classification, we found higher proportions of cases with sufficient DC/DC_leu_-generation using WB than MNC, letting DC/DC_leu_-generation with standard protocols appear more reliable from WB than MNC.

Overall, we found the standard protocols perform better in a WB-environment than in the MNC-environment they were originally designed for.

### 3.3. New Kits Specifically Designed for WB

We developed nine new DC/DC_leu_-generating protocols (kits) specifically designed for the generation of DC/DC_leu_ from leukaemic WB. The new kits were derived from the standard protocols and composed of one to three response modifiers, including GM-CSF, IFNa, TNFa, PGE_1_, OK-432 and Ca-Iono.

We compared the performance of the new kits to their respective standard protocols and found Kit-F generated significantly higher, Kit-A, -C, -D, -G, -I and -K generated equal and Kit-E and -H generated significantly lower frequencies of DC/DC_leu_ from WB compared to their respective standard protocol. Frequencies of mature DC were comparable between kits and standard protocols (Figure 3). Assessing kits using the DC/DC_leu_-classification showed a similar picture: we found Kit-F, -G (when compared to Mcm), -D, -I, -A and -K achieved higher proportions of cases with excellent DC/DC_leu_-generation and Kit-C, -E, -G (when compared to Pici) and -H achieved lower proportions of cases with excellent DC/DC_leu_-generation compared to their respective standard protocol (Figure 4).

Overall, most kits performed comparable or even better compared to their respective standard protocol. Only Kit-E and -H performed worse in comparison.

### 3.4. Ranking of Kits

We further ranked kits according to their performance in generating DC/DC_leu_. Ranking kits with the class-ranking (based on proportions of cases with excellent, good and satisfactory generation of DC/DC_leu_), we found Kit-K and -I achieved the best results (Figure 5A, Table 2). Ranking kits with the best-ranking (based on proportions of cases with the best and second-best generation of DC), we found Kit-F and -A = -D = -I achieved the best results (Figure 5B, Table 3).

Overall, we found Kit-K, -I, -A, -D and -F containing GM-CSF combined with OK-432, PGE_2_, TNFa and/or Ca-Iono the best performing kits, initiating sufficient DC/DC_leu_-generation in most cases. Interestingly, although GM-CSF has been shown to mediate differentiation, activation and maturation of dendritic cells [26,27], GM-CSF on its own, as in Kit-G, did not show sufficient DC/DC_leu_ generation, which is in line with previous findings [42,43,44]. DC/DC_leu_-generation with GM-CSF seems to be dependent on the synergetic effects of additional response modifiers, like OK-432, PGE_2_ or Ca-Iono, that further enhance dendritic differentiation and proliferation and moreover stimulate activation and maturation, properties essential for mediating a comprehensive immune response [42,45,46]. Of note, a combination of GM-CSF with two other response modifiers, OK-432 and PGE_2_, as in Kit-D, showed no advantage over a combination of GM-CSF with either OK-432 or PGE_2_, as in Kit-I and -K. Saturation of the DC/DC_leu_-generation-pathway might be at play here. The Ca-Iono containing Kit-F showed good results in line with previous findings [47,48]. Mentionable, Ca-Iono containing protocols like Kit-F hold the significant advantage of reduced culture times of two days without constrained success of DC/DC_leu_-generation and T-cell stimulation through generated DC/DC_leu_; however, such protocols have constrained overall viability of DC/DC_leu_ compared to other protocols [47,48]. When comparing the TNFa containing Kit-C to Kit-A, we found Kit-C performed worse than -A. This is dissenting with other findings that suggest PGE_2_ to further enhance the effect GM-CSF together with TNFa [46,49]. The IFNa containing Kit-E and -H showed poor performance. This might be surprising at first as IFNa is well-known for its anti-tumour effects and its role in the treatment of several solid and haematological malignancies [50,51,52,53]. In AML, IFNa has been shown to directly inhibit blast proliferation and moreover stimulate blast apoptosis [51,54]—properties that yet come amiss when aiming to convert myeloid leukaemic blasts into DC_leu_, hampering the overall DC/DC_leu_-generating process.

### 3.5. Selection of Kits for (Potential) Treatment of Patients

In regard to future clinical applications, the development of kits has to consider not only the potential of kits but also their biocompatibility and applicability. Of the nine new kits we developed, Kit-K, -I, -A, -D and -F showed the best performance, thus qualifying for further in-depth evaluation. As TNFa and Ca-Iono were no longer approved for human systemic treatment, we had to exclude Kit-A and -F from further considerations. As Kit-D constitutes a combination of Kit-I and -K with no advantage to the individual protocols but with a more intricate composition of three response modifiers in contrast to two response modifiers of the individual protocols, we also excluded Kit-D from further considerations. Lastly, as parallel studies with PGE_1_ showed PGE_2_ and PGE_1_ to have comparable effects on the generation of DC/DC_leu_, we introduced a new Kit-M, derived from Kit-K, consisting of GM-CSF and PGE_1_, for further evaluation [37].

Overall, we selected Kit-I, -K and -M as the most promising kits and further scrutinised their capacity to generate (mature) DC/DC_leu_ as well as—and most importantly—their potential to initiate anti-leukaemic cytotoxicity through generated DC/DC_leu_. All three kits were able to generate significantly higher frequencies of DC/WB and DC_leu_/WB compared to the control (Figure 6). Notably, Kit-generated DC/DC_leu_ hereby consisted of significant frequencies of mature DC. Both class- and order-ranking determined Kit-I to be superior to Kit-M and -K (Figure 7A,B and Table 4 and Table 5). All three kits were able to improve the anti-leukaemic cytotoxicity of immunoreactive cells through generated DC/DC_leu_ in most of the cases. Interestingly, some cases achieved an improvement in lysis after 3 h and some cases only after 24 h, whereas the average improvement in lysis was the highest in MLC^Kit-K^ and MLC^Kit-M^ after 3 h and in MLC^Kit-I^ after 24 h. This can be explained by the different mechanisms by which immunoreactive cells exert cytotoxicity: the early and fast-acting perforin-granzyme pathway (as likely seen in MLC^Kit-K^ and MLC^Kit-M^) and the late and slow-acting Fas/FasL pathway (as likely seen in MLC^Kit-I^), which can run separately or synergistically [55,56]. Overall, pooling the best anti-leukaemic cytotoxicity after 3 or 24 h, DC/DC_leu_ generated with Kit-I appear to be the most efficient in stimulating blast-lytic activity, followed by Kit-M and -K (Figure 8).

Taken together, all three kits were able to generate significant amounts of (mature) DC/DC_leu,_ which have the competence to regularly mediate anti-leukaemic cytotoxicity. Kit-I hereby performed the best, followed by Kit-M and -K, regarding the class- and best-ranking as well as the overall best-achieved improvement in blast lysis. We thus perceive Kit-I and -M to have the highest potential in generating DC/DC_leu_ and stimulating anti-leukaemic activity.

### 3.6. Ongoing Kit Studies

Kit-I, -K, -M have shown auspicious results. On the way to successful in vivo treatment of patients with AML, kits though have to undergo further investigations regarding their performance and toxicity.

In this study, we could show that kits are competent to generate (mature) DC/DC_leu_ that regularly initiate anti-leukaemic activity. We could confirm these results in multiple studies [24,37]. Furthermore, we could enlighten the leukaemia-specific activity of innate and adaptive immunoreactive cells on a single-cell level by analysing their IFNy secretion profiles and correlating them with the overall achieved anti-leukaemic cytotoxicity, thereby being able to display (indirectly and directly) participating immunoreactive cells of DC/DC_leu_-mediated leukaemia-specific and anti-leukaemic immune responses: levels of IFNy secreting innate (NK cells, CIK cells, iNKT cells) and adaptive (T-cells) immune cells were significantly higher in MLC^Kits^ compared to MLC^Control^, with frequencies of IFNy secreting T^CD3+^, T^CD4+^, T^CD8+^ and NK^56+^ cells positively correlating with the overall achieved anti-leukaemic activity [24]. When assessing compositions of DC/DC_leu_ stimulated T-enriched immunoreactive cells, we found lower frequencies of regulatory T-cells (T_reg_) in MLC^Kits^ compared to MLC^Control^ [57]; an important finding as the success of immunomodulation is directly affected by the T_regs_ immunosuppressive disposition [58]. A milestone in drug development though not only is the verification of anti-leukaemic effects but also the exclusion of pro-leukaemic effects. We thus investigated the proliferation of blasts in relation to kit treatment but found no induction of blast proliferation through kit treatment in this regard [59]. Of note, DC/DC_leu_-generation and -functioning was comparable under normoxic (21% oxygen) and hypoxic (10% oxygen, resembling the in vivo situation) conditions [57].

Ultimately, DC/DC_leu_-based immunotherapy needs to be transferred from in vitro to in vivo settings to further assess its value in the treatment of AML. We thus extended our preclinical studies from in vitro to in vivo animal trials to gather more information about the kits’ efficiency and toxicity. Kit treatment (Kit-M) of leukaemically diseased rats resulted in the generation of DC/DC_leu_ without induction of blast proliferation and consecutively in an improved anti-leukaemic (blast-lytic) activity [60], displaying in vivo functionality of kits. For future studies, we are now focusing on conducting kit studies in patients with refractory AML, and striving to replicate the promising in vitro and in vivo results of DC/DC_leu_-based immunotherapy in clinic.

## 4. Materials and Methods

### 4.1. Sample Collection and Preparation

Samples were collected in the form of heparinised whole blood from patients in acute stages of AML and MDS after obtaining written consent and in accordance with the World Medical Association Declaration of Helsinki and the ethics committee of the Ludwig-Maximilian-University Hospital (vote no 33905). Samples were provided by the university hospitals of Munich, Tuebingen, Oldenburg and Augsburg.

MNC were isolated from the patients’ blood samples using density gradient centrifugation (Biocoll-Separating-Solution, Biochrom, Berlin, Germany). T-cells were isolated from MNC by CD3+ positive MACS microbead and column-based immunomagnetic cell separation technology (Miltenyi Biotec, Bergisch Gladbach, Germany) according to the manufacturer’s instructions. MNC and T-cells, unless directly used, were frozen with 70% RPMI-1640 medium (Biochrom), 20% human serum (Health Care Europa GmbH, Vienna, Austria) and 10% dimethyl sulfoxide (Sigma Aldrich Chemie GmbH, Steinheim, Germany), stored at −80 °C and thawed when required.

### 4.2. Patients’ Characteristics

Blood samples were obtained from patients in acute phases of AML (*n* = 65) and MDS (*n* = 2) with an average age of 54.3 (range 21–88) years and a female-to-male ratio of 1:1.3. Patients were classified based on the French-American-British (FAB) classification (M0-M5), the aetiology (primary AML, secondary AML), the stage of disease (diagnosis, persistence, relapse, relapse after SCT), the blast phenotype and frequency. An overview is given in Table 6.

### 4.3. Flow Cytometry

Flow cytometric analyses were performed to assess and quantify frequencies, phenotypes and subsets of leukaemic blasts, DC and T-cells. In cases of aberrant expression of T, B, NK or monocytic antigens, proportions were not included in the analyses. Abbreviations of all cell types are given in Table 7.

Therefore, cells were stained with various monoclonal antibodies (moAbs) labelled with fluorescein isothiocyanate (FITC)^a^, phycoerythrin (PE)^b^, phycoerythrin-cyanine-7 tandem conjugate (PE-Cy7)^c^ or allophycocyanin (APC)^d^. Antibodies were provided by Becton Dickinson (Heidelberg, Germany) (CD1^ab^, CD1b^a^, CD14^c^, CD15^d^, CD71^c^, CD206^d^, 7AAD^c^, CCR7^c^), Beckmann Coulter (Krefeld, Germany) (CD1^ab^, CD3^a^, CD19^c^, CD33^d^, CD56^b^, CD80^b^, CD117^b,d^, CD206^b^, CD34^a,c^,CD65^a^, CD83^a^) and Thermo Fisher Scientific (Darmstadt, Germany) (CD1^ab^,CD13^c^,CD34^d^ and CD86^a^).

Cell staining was performed by incubating cells with corresponding moAbs for 15 min in the dark using a staining medium containing 80% PBS (Biochrom) and 20% FCS (Biochrom). In the case of WB samples, erythrocytes were lysed prior to staining using a lysing buffer (Becton Dickinson) according to the manufacturers’ instructions. Cells were analysed with the fluorescence-activated cell sorting flow cytometer FACS Calibur (Becton Dickinson) and the acquisition and analysis software CellQuestPro (Becton Dickinson). Isotype controls were conducted according to the manufacturer’s instructions.

### 4.4. Dendritic Cell Culture (DCC)

DC/DC_leu_ were generated from MNC and WB following refined DC/DC_leu_-generating protocols using specific response modifiers, including Ca-Ionophore A23187 (Ca-Iono) (Sigma Aldrich Chemie GmbH), FLT3-ligand (FL) (PromoCell, Heidelberg, Germany), granulocyte-macrophage-colony-stimulating-factor (GM-CSF) (Sanofi-Aventis, Frankfurt, Germany), interferon-alpha (IFNa) (Essex Pharma, Munich, Germany), interleukin-1-beta (IL-1b) (Cell Concepts, Umkirch, Germany), interleukin-4 (IL-4) (PeproTech, Berlin, Germany), interleukin-6 (IL-6) (Cell Concepts); picibanil (OK-432) (Chugai Pharmaceutical, Kajiwara, Japan); tumor-necrosis-factor-alpha (TNFa) (Cell Concepts), prostaglandin-E_1_ (PGE_1_) (PeproTech) and prostaglandin-E_2_ (PGE_2_) (PeproTech) (Table 8).

For MNC-based cultures (as used for Ca, Mcm, Pici), 7 × 10^5^ MNC/mL (Ca), 2.5 × 10^6^ MNC/mL (Mcm) or 1 × 10^6^ MNC/mL (Pici) were cultured in 12-multiwell-culture-plates (Thermo Fisher Scientific) and diluted with 2 mL x-vivo-15-medium (Lonza, Basel, Switzerland). For WB-based cultures (as used for Ca, Mcm, Pici, Kit-A, -C, -D, -E, -F, -G, -H, -I, -K and -M), 0.5–2 mL WB were cultured in 12-multiwell-culture-plates and diluted with 0.5–2 mL x-vivo-15-medium. Response modifiers were added to all cultures according to the protocols. One culture without added response modifiers severed as control. Cells were cultured under standard conditions at 21% O_2_, 5% CO_2_ and 37 °C, and harvested following the protocols. A half-medium-exchange was carried out after 7–8 days in Mcm-cultures.

**Figure 9:**
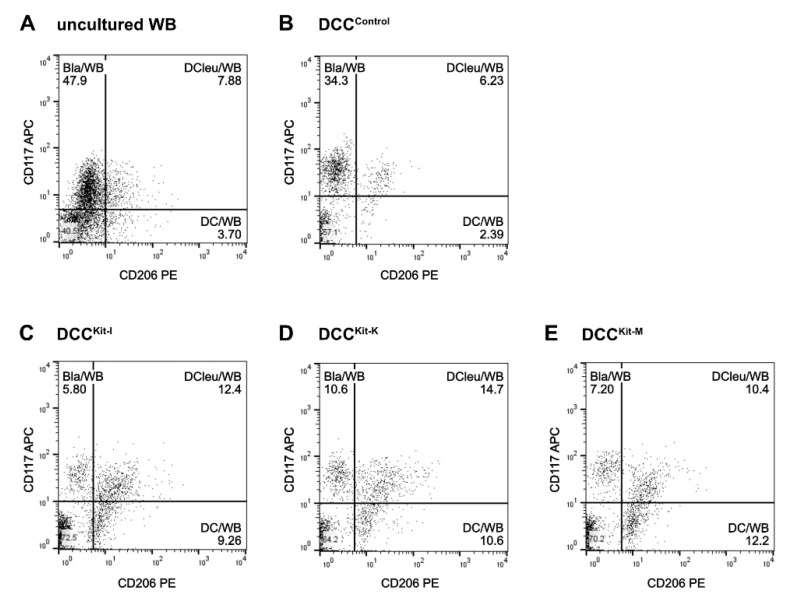
Flow cytometric analyses of DC_leu_. Examplary plots show DC_leu_, characterised by the coexpression of the blast marker CD117 and the DC marker CD206, in uncultured WB (**A**) and after kit treatment with Kit-I (DCC^Kit-I^) (**C**), Kit-K (DCC^Kit-K^) (**D**), Kit-M (DCC^Kit-M^) (**E**) as well as the Control (DCC^Control^) (**B**).

Flow cytometric analyses of (mature) DC and DC_leu_ (subgroups) were performed following a refined gating strategy [10,11,17]. DC_leu_ were analysed by the co-expression of at least one blast marker (CD15, CD34, CD65, CD117), including lineage-aberrant markers (CD56) and one dendritic marker (CD80, CD83, CD86, CD206, CD209) that had not been expressed on naive blasts (Figure 9). The premise for DC subgroup analyses was the presence of ≥10% DC/cells.

To analyse the competence of DC/DC_leu_-generating protocols to generate DC/DC_leu_, we developed a refined classification system, the DC/DC_leu_-classification, based on frequencies of DC/cells and DC_leu_/cells generated. DC/DC_leu_-generation was classified as ‘sufficient’, further subdivided into ‘excellent’, ‘good’ and ‘satisfactory’ or as ‘insufficient’, as given in Table 1.

### 4.5. Mixed Lymphocyte Culture (MLC)

DC/DC_leu_ from WB-cultures were used to stimulate T-cell enriched immunoreactive cells. Therefore, a fraction of DCC containing 25 × 10^3^ DC/DC_leu_ and 1 × 10^6^ T-cells were co-cultured in 24-multiwell-culture-plates (Thermo Fisher Scientific) and diluted in 1 mL RMPI-1640 containing 100 U/mL penicillin. 50 U/mL interleukin-2 (IL-2) (PeproTech) were added to all cultures on day 0 and days 2–3. Cells were cultured following the protocols under standard conditions at 21% O_2_, 5% CO_2_ and 37 °C. A half-medium exchange was carried out after 2–3 days.

### 4.6. Cytotoxicity Fluorolysis Assay (CTX)

The blast lytic activity of DC/DC_leu_ stimulated T-cell enriched immunoreactive cells in MLC from WB-cultures was analysed using a cytotoxicity fluorolysis assay (CTX). Therefore, a fraction of MLC containing 1 × 10^6^ T-cells (effector cells) was co-cultured with 1 × 10^6^ thawed autologous leukaemic blasts (target cells) for 3 and 24 h at 37 °C, 21% O_2_ and 5% CO_2_. Target cells were stained with blast specific moAbs before culture and with 7AAD and a particular number of fluorosphere beads (Beckman Coulter) after culture when harvested. As a control, effector and target cells were cultured under the same conditions but separated and only combined prior to flow cytometric analyses.

Flow cytometric analyses were performed using a refined gating strategy [21], whereas the achieved blast lytic activity was defined as the percentual difference of viable target cells (blasts) between the effector-target-co-culture and the control.

### 4.7. Statistical Methods

Data are presented as mean ± standard deviation (SD). Statistical analyses were implemented using the two-tailed *t*-test. Significance was defined as ‘not significant’ (n.s.) with *p* values > 0.10, as ‘borderline significant’ with *p* values 0.10 to 0.05, as ‘significant’ with *p* values 0.05 to 0.005 and as ‘highly significant’ with *p* values < 0.005. All statistical analyses and figures were implemented using Excel 2013 (Microsoft, Redmond, WA, USA), SPSS (IBM, Armonk, NY, USA) and Prism 9 (GraphPad Software, San Diego, CA, USA).

## 5. Conclusions

In this study, we developed DC/DC_leu_-generating protocols (kits; comprising one to three response modifiers) specifically designed for the application in a WB-environment. We assessed kits based on their capacity to generate mature DC/DC_leu_ from leukaemic myeloid blasts and to mediate DC/DC_leu_-based anti-leukaemic immunity. Ultimately, we identified Kit-I, -K and -M as the best performing kits in vitro, qualifying for further evaluation in vitro and vivo. In regard to future clinical applications, these findings are of particular importance as they are the basis for establishing clinical protocols for successful DC/DC_leu_-based immunotherapy of AML patients in vivo.

## 6. Patents

Modiblast Pharma GmbH (Oberhaching, Germany) holds the European Patent ‘EP 3,217,975 B1′ and the US Patent ‘US 10,912,820′, in which H.M.S. is involved.

## Figures and Tables

**Table 1:** Classification of DC- and DC_leu_-generation.

Subcategories		DC/Cells		DC_leu_/Cells
**Sufficient**	**Excellent**	≥15%	*and*	≥10%
	**Good**	≥15%	*and*	<10% and ≥5%
		or		
		<15% and ≥10%	*and*	≥ 10%
	**Satisfactory**	<15% and ≥10%	*and*	<10% and ≥5%
**Insufficient**		<10%	*and/or*	<5%

DC/DC_leu_-generation is assessed with respect to frequencies of generated DC/cells and/or DC_leu_/cells. Results were divided in sufficient, further subdivided in excellent, good and satisfactory and insufficient.

**Table 2:** Class-ranking of all kits.

Rank	Ranking 1 *Excellent*	Ranking 2 *Excellent, Good*	Ranking 3 *Excellent, Good, Satisfactory*		Ranking 4 *Combined*
1	I	K	K	→	K
2	K	I	I	→	I
3	F	A	A	→	A
4	A	F	F	→	F
5	D	D	D	→	D
6	G	C	C	→	C
7	C	G	G	→	G
8	E	E	E	→	E
9	H	H	H	→	H

Kits (A, C, D, E, F, G, H, I, K) were ranked based on the proportions of cases with excellent, good, and/or satisfactory generation of DC/WB and DC_leu_/WB.

**Table 3:** Best-ranking of all kits.

Rank	Ranking 1 *Best*	Ranking 2 *Best, Second-Best*		Ranking 3 *Combined*
1	F	F	→	F
2	A	I	→	A = D = I
3	D	D	→	
4	I	A	→	
5	E	E	→	E
6	C	K	→	K = C
7	K	C	→	
8	G	G	→	G
9	H	H	→	H

Kits (A, C, D, E, F, G, H, I, K) were ranked based on the proportions of cases with best and/or second-best generation of DC/WB.

**Table 4:** Class-ranking of selected kits.

Rank	Ranking 1 *Excellent*	Ranking 2 *Excellent, Good*	Ranking 3 *Excellent, Good, Satisfactory*		Ranking 4 *Combined*
1		I	I	→	I
2	I, M	K	M	→	M
3	K	M	K	→	K

Selected kits (I, K, M) were ranked based on the proportions of cases with excellent, good and/or satisfactory generation of DC/WB and DC_leu_/WB.

**Table 5:** Best-ranking of selected kits.

Rank	Ranking 1 *Best*	Ranking 2 *Best, Second-Best*		Ranking 3 *Combined*
1	I	I	→	I
2	M	K	→	M = K
3	K	M	→	

Selected kits (I, K, M) were ranked based on the proportions of cases with best and/or second-best generation of DC/WB.

**Table 6:** Patients’ characteristics.

Patient No.	Age, Sex	Stage	FAB Type	Blast Phenotype (CD)	IC Blasts (%)	Source	Conducted Experiments
**AML**							
1172	24, m	dgn	p-M0	117,13,33,34	92	MNC, WB	DCC
993	71, m	dgn	p-M1	34,13,33,117	95	MNC, WB	DCC
1289	65, w	dgn	p-M1	34,33, 56,117	60	WB	DCC
1300	24, w	dgn	p-M1	34,13,33,65,117	75	WB	DCC, CTX
1190	72, m	dgn	p-M2	117,33,34	28	MNC, WB	DCC
1292	44, w	dgn	p-M2	117,13,33,34	78	WB	DCC, CTX
1295	40, m	dgn	p-M2	34,13,33,117	45	WB	DCC
1225	48, m	dgn	p-M3	117,13,33	77	MNC, WB	DCC
1327	61, m	dgn	p-M4	117,7,13,33,65	75	WB	DCC
1453	54, w	dgn	p-M4	15,14,33,64,56	52	WB	DCC, CTX
1459	54, m	dgn	p-M4	56,4,11c,11b,33,38,64	14	WB	DCC, CTX
1460	78, w	dgn	p-M4	15,34,117	68	WB	DCC, CTX
1430	79, m	dgn	p-M5	34,117,13,33	70	WB	DCC, CTX
1432	34, m	dgn	p-M5	34,13,33,64	81	WB	DCC, CTX
1447	21, m	dgn	p-M5	33,56,45	65	WB	DCC, CTX
1443	64, m	dgn	p-M?	34,117,13,33	50	WB	DCC
1452	44, m	dgn	p-M?	34,117,33,13,45	55	WB	DCC, CTX
1050	32, w	dgn	s-M1	117,14,13,33	90	MNC, WB	DCC
1144	32, m	dgn	s-M1	34,13,19,56,117	60	MNC, WB	DCC
1204	72, m	dgn	s-M2	117,13,56	43	MNC, WB	DCC
1131	53, w	dgn	s-M4	117,33	15	WB	DCC
1207	56, w	dgn	s-M4	117,4,13,15,33,34	10	MNC, WB	DCC
1442	73, w	dgn	s-M4	117,33,61,138	14	WB	DCC, CTX
1426	61, w	dgn	s-M5	34,117,13,33,64	93	WB	DCC, CTX
1439	61, w	dgn	s-M5	34,117,13,33	17	WB	DCC
999	51, w	dgn	s-M?	34,13,33,65,117	14	MNC, WB	DCC
1056	27, m	dgn	s-M?	117,13,15,33,34	42	MNC, WB	DCC
1165	59, m	dgn	s-M?	34,13,33,117	46	MNC, WB	DCC
1194	72, m	dgn	s-M?	34,33,117	18	MNC, WB	DCC
1196	39, w	dgn	s-M?	117,33	11	MNC, WB	DCC
1226	69, m	dgn	s-M?	117,33,34	65	MNC, WB	DCC
1385	82, m	dgn	s-M?	34,13,56,117	23	WB	DCC, CTX
1434	61, w	dgn	s-M?	34,117,7,13,33,56,64	61	WB	DCC, CTX
1449	78, m	dgn	s-M?	15,65,4,45,64	62	WB	DCC, CTX
1454	60, w	dgn	s-M?	34,117,20,61	33	WB	DCC
1024	39, m	pers	p-M2	34,33,117	88	MNC, WB	DCC
1218	54, m	pers	p-M2	33,56,13	30	MNC, WB	DCC
1201	60, w	pers	p-M5b	34,4,13,14,15,33,56,6,5	63	WB	DCC
1320	66, m	pers	s-M6	34,65,117	15	WB	DCC, CTX
1310	77, w	pers	s-M?	117,33,34,56	52	WB	DCC
1011	88, w	rel	p-M1	34,13,33,65,117	88	MNC, WB	DCC
1127	61, w	rel	p-M1	34,13,33,117	46	MNC, WB	DCC
1138	24, m	rel	p-M1	34,4,14,33,56,65,117	50	MNC, WB	DCC
1222	40, m	rel	p-M1	117,33,34,56	50	MNC, WB	DCC
1080	37, w	rel	p-M2	34,4,13,33,117	34	MNC, WB	DCC
1123	70, m	rel	p-M2	34,13,33,117	47	MNC, WB	DCC
1205	60, m	rel	p-M2	34,2,13,33,117	67	MNC, WB	DCC
1206	74, w	rel	p-M2	34,4,33,117	27	MNC, WB	DCC
1243	34, m	rel	p-M2	34,2,13,33,117	32	MNC, WB	DCC
1376	52, w	rel	p-M2	34,33,117	60	WB	DCC, CTX
1387	63, m	rel	p-M2	34,13,33,117	37	WB	DCC, CTX
1001	60, w	rel	p-M4	34,33,117	35	MNC, WB	DCC
1017	67, m	rel	p-M4	34,33,117	34	MNC, WB	DCC
1018	40, w	rel	p-M4	34,15,33,64,117	17	MNC, WB	DCC
1143	46, w	rel	p-M4	117,2,7,13,34,65	75	MNC, WB	DCC
998	67, m	rel	p-M5a	34,4,64,117	92	MNC, WB	DCC
1263	40, m	rel	p-M5	34,19,33,117	18	WB	DCC
1375	44, w	rel	p-M5	34,15,33,117	35	WB	DCC, CTX
1303	64, w	rel	s-M4	34,4,13,33,14,56,65	65	WB	DCC, CTX
1286	21, m	rel	s-M5	117,33,34	35	WB	DCC, CTX
987	61, m	rel	s-M?	34,13,33,117	21	MNC, WB	DCC
1183	36, w	rel	s-M?	34,33,117	33	MNC, WB	DCC
1307	54, m	rel	s-M?	117,13,15,19,33,34,65	30	WB	DCC, CTX
1386	57, m	rel	s-M?	34,33,117	78	WB	DCC, CTX
**MDS**							
1433	59, m			34,117,14,33	14	WB	DCC, CTX
1306	42, m			117,13,33,34	11	WB	DCC, CTX

AML acute myeloid leukaemia; MDS myelodysplastic syndrome; m male; f female; pAML primary AML; sAML secondary AML; FAB french-american-british classification; M? FAB type not classified; dgn diagnosis; pers persistance; rel relapse; IC blasts immunocytologically determined blasts; WB whole blood; MNC mononuclear cells; DCC dendritic cell culture; CTX cytotoxicity fluorolysis assay; bold markers were used to detect DC_leu_.

**Table 7:** Cell types.

Cell Types	Cell Abbreviation	Surface Marker	Referred To	Abbreviation	Reference
**Blasts**	Bla	Bla^+^ (CD15^+^, CD34^+^, CD65^+^, CD117^+^)	MNC or WB WB	Bla/cells Bla/WB	[10]
**Dendritic Cells**	DC	DC^+^ (CD80^+^, CD83^+^, CD86^+^, CD206^+^, CD209^+^,)	MNC or WB WB	DC/cells DC/WB	[10]
**Leukaemia Derived** **Dendritic Cells**	DC_leu_	DC^+^Bla^+^	MNC or WB WB DC	DC_leu_/cells DC_leu_/WB DC_leu_/DC	[10]
**Mature Migratory** **Dendritic Cells**	DC_mig_	DC^+^CCR7^+^	DC	DC_mig_/DC	[12]

Cell types and their surface marker combinations for flow cytometric staining and analysis. WB whole blood; MNC mononuclear cells.

**Table 8:** DC/DC_leu_-generating protocols.

DC/DC_leu_-Protocol	DC/DC_leu_-Source	Composition (Total) and Time Added (d)	Culture Time (d)	References
**Ca**	MNC WB	Ca-Iono 375 ng/mL (d1) IL-4 250 U/mL (d1)	3–4	[11,19]
**Mcm**	MNC WB	GM-CSF 800 U/mL (d1, d4-5) TNFa 200 U/mL (d7-8) PGE_2_ 1 μg/mL (d7-8) IL-1b 5 ng/mL (d7-8) IL-4 500 U/mL (d1, d4-5) IL-6 150 ng/mL (d7-8) FL 40 ng/mL (d, d4-5)	10–14	[11,19]
**Pici**	MNC WB	GM-CSF 500 U/mL (d1) OK-432 10 μg/mL (d7-8) IL-4 250 U/mL (d1)	9–11	[11]
**Kit-A**	WB	GM-CSF 800 U/mL (d1, d2-3) TNFa 200 U/mL (d1, d2-3)	7–10	European Patent EP 3217975 B1
**Kit-C**	WB	GM-CSF 800 U/mL (d1, d2-3) TNFa 200 U/mL (d, d2-3) PGE_2_ 1 µg/mL (d1, d2-3)	7–10	
**Kit-D**	WB	GM-CSF 800 U/mL (d1, d2-3) OK-432 10 µg/mL (d1, d2-3) PGE_2_ 1 µg/mL (d1, d2-3)	7–10	
**Kit-E**	WB	GM-CSF 800 U/mL (d1, d2-4, d7-8) IFNa 500 U/mL (d1, d2-4, d7-8)	8–11	[19]
**Kit-F**	WB	Ca-Iono 250 ng/mL (d1, d2-3) GM-CSF 800 U/mL (d1, d2-3)	3–4	European Patent EP 3217975 B1
**Kit-G**	WB	GM-CSF 800 U/mL (d1, d2-3)	7–10	
**Kit-H**	WB	IFNa 500 U/mL (d1, d2-4, d7-8)	7–10	[19]
**Kit-I**	WB	GM-CSF 800 U/mL (d1, d2-3) OK-432 10 μg/mL (d1, d2-3)	7–10	European Patent EP 3217975 B1 US Patent US 10,912,820
**Kit-K**	WB	GM-CSF 800 U/mL (d1, d2-3) PGE_2_ 1 μg/mL (d1, d2-3)	7–10	European Patent EP 3217975 B1
**Kit-M**	WB	GM-CSF 800 U/mL (d1, d2-3) PGE_1_ 1 μg/mL (d1, d2-3)	7–10	European Patent EP 3217975 B1 US Patent US 10,912,820

Ca-Iono Ca-Ionophore A23187; FL FLT3-ligand; GM-CSF granulocyte-macrophage-colony-stimulating-factor; IFNa interferon-alpha; IL-1b interleukin-1-beta; IL-4 interleukin-4; IL-6 interleukin-6; OK-432 picibanil; TNFa tumor-necrosis-factor-alpha; PGE_1_ prostaglandin-E_1_; PGE_2_ prostaglandin-E_2_; d day.

## Data Availability

The data presented in this study are available on request from the corresponding author.
